# Workplace Coping Strategies Among the Nursing Workforce: A Cross-Sectional Study From an Indian Perspective

**DOI:** 10.7759/cureus.95812

**Published:** 2025-10-31

**Authors:** Hepsi Bai Joseph, Vinitha Ravindran

**Affiliations:** 1 Pediatrics, College of Nursing, All India Institute of Medical Sciences, Bhubaneswar, Bhubaneswar, IND; 2 Nursing, Christian Medical College Vellore, Vellore, IND

**Keywords:** approach, coping, india, job stress, nurses, nursing workforce, strategies, stressors

## Abstract

Background

Nurses frequently encounter stress in hospital settings due to high patient loads, emotional demands, and complex work environments. Effective coping strategies are crucial for maintaining mental health and job performance. This study aimed to assess the coping strategies adopted by nurses and to determine the associated factors with nurses' coping.

Methods

A descriptive cross-sectional study was conducted among 400 hospital nurses to assess their coping strategies working at a tertiary care hospital in South India. Data were collected using BRIEF COPE inventory. Statistical analysis employed both descriptive and inferential methods to identify common coping mechanisms and assess associated factors with nurses' coping.

Results

Nurses reported a mean coping score of 61.6±12.6, with a higher use of approach coping (27.8±4.4) compared to avoidance coping (22.6±4.1). Significant associations were found between coping and sociodemographic variables (age, children, income, marital status) as well as professional factors (experience) (p < 0.05).

Conclusion

The study highlights nurses' reliance on approach coping strategies, influenced by sociodemographic and professional variables. Enhancing coping mechanisms through targeted interventions can improve job satisfaction, reduce stress, and enhance performance. Policymakers should implement supportive workplace policies, offer stress management programs, and promote work-life balance to ensure nurses' well-being and optimal healthcare delivery.

## Introduction

Nursing is an indispensable pillar of the healthcare system, requiring a unique combination of technical expertise, emotional resilience, and interpersonal skills. The role of nurses extends beyond routine patient care to include managing emergencies, coordinating with multidisciplinary teams, and addressing the psychological and emotional needs of patients and families. However, the demanding nature of the profession often places nurses at risk of experiencing significant job stress. This stress, if unaddressed, can lead to adverse physical, emotional, and professional consequences, including burnout, compassion fatigue, and attrition [1]. The exploration of coping, coping strategies, and associated factors in managing job stress among nurses is thus vital to improving their well-being and sustaining high-quality patient care.

Coping refers to individuals' cognitive and behavioral efforts to manage stressors perceived as taxing or exceeding their resources [2]. For nurses, stress can stem from multiple sources, such as heavy workloads, shift duties, inadequate staffing, exposure to suffering and death, and workplace conflicts [3]. Effective coping mechanisms enable nurses to mitigate the adverse effects of stress, maintain their mental health, and perform their duties efficiently. Conversely, maladaptive coping strategies can exacerbate stress, impair job performance, and compromise patient safety [4]. Coping strategies are broadly categorized into problem-focused and emotion-focused approaches [2]. Problem-focused coping involves addressing the root causes of stress through strategies such as time management, delegation, and seeking professional support. Emotion-focused coping, on the other hand, aims to alleviate the emotional distress associated with stressors through methods such as relaxation, mindfulness, and seeking social or spiritual support. Nurses often use a combination of these strategies, depending on the nature of the stressor and their individual and organizational context [5]. Adaptive coping strategies have far-reaching benefits for nurses. They help reduce stress, prevent burnout, and enhance job satisfaction and resilience. Mindfulness-based interventions, stress management programs, and peer support groups have been shown to improve nurses’ coping skills and overall well-being [6]. Additionally, effective coping has a positive impact on patient outcomes, as nurses who manage stress well are more likely to provide high-quality care [7]. Conversely, maladaptive coping strategies, such as avoidance and substance use, can worsen stress levels and lead to adverse outcomes. A study by Khamisa et al. found that nurses who relied on avoidance coping were more likely to experience burnout and psychological distress [4]. These findings underscore the importance of promoting adaptive coping mechanisms through targeted interventions.

Several individual, professional, and organizational factors influence nurses' coping mechanisms. Socio-demographic variables such as age, gender, marital status, educational background, and years of experience significantly affect coping strategies [8]. For instance, older nurses and those with greater experience often exhibit more effective coping skills, drawing on their accumulated knowledge and resilience [9].

Professional factors, including job type, work environment, and shift patterns, also play a critical role. Nurses working in high-intensity settings such as ICUs and emergency departments face unique stressors that demand specific coping strategies [3]. Organizational factors, such as the availability of resources, perceived administrative support, and workplace culture, further shape nurses' coping behaviors. A supportive work environment enhances adaptive coping mechanisms and reduces the likelihood of burnout [10].

In India, nurses represent the largest group of healthcare professionals and play a pivotal role in delivering patient-centered care across diverse healthcare settings. However, they frequently experience high levels of occupational stress due to heavy workloads, inadequate staffing, long working hours, hierarchical organizational structures, and limited professional autonomy. Studies have consistently reported that job-related stress negatively impacts nurses’ physical and psychological well-being, job satisfaction, and the quality of patient care. Coping strategies commonly employed by Indian nurses, such as emotion-focused coping, spiritual coping, and social support, reflect the strong influence of cultural values, collectivism, and the emphasis on endurance and duty within the Indian nursing profession. Despite the growing body of research, several gaps persist. Most studies have examined the interplay between workplace environment, institutional support, and individual coping behaviors within the cultural context of Indian nursing practice. There remains a need for studies that not only quantify occupational coping but also explore contextually relevant, resilience-building strategies suited to the Indian healthcare system. The present study addresses these gaps by examining the coping strategies among nurses working in tertiary care hospitals in India, thereby contributing empirical evidence specific to the Indian institutional context. By focusing on the cultural dimensions of coping and the institutional factors that influence stress management, this study aims to provide insights that can inform policy, education, and workplace interventions tailored to the Indian nursing workforce. Hence, the present study was conducted with the objective of assessing the coping strategies adopted by nurses and to determine the professional and personal factors associated with coping.

## Materials and methods

Methodology

A cross-sectional study was conducted to assess the coping strategies adopted by nurses working at a selected tertiary-level teaching hospital in South India. The study was conducted in a state government tertiary care teaching hospital in Kanyakumari district, Tamil Nadu, which provides comprehensive healthcare services, including general, specialty, and super-specialty care. The institution also serves as a major training center for undergraduate and postgraduate nursing and medical students, offering a dynamic clinical environment characterized by high patient turnover, multidisciplinary teamwork, and demanding workloads. This setting was chosen as it reflects the typical working conditions, organizational structure, and cultural environment of tertiary-level public hospitals in India, thereby providing an appropriate context for examining occupational stress and coping strategies among nursing professionals. The first author was affiliated with the Kanyakumari Government Medical College to conduct the research study. The second author was the research supervisor for the study.

Sampling

A total enumeration sampling method was adopted to collect information from all eligible nurses working in the selected tertiary-level teaching hospital. In this method, every individual meeting the inclusion criteria within the defined population is included in the study, ensuring complete coverage and minimizing sampling bias. This approach was chosen because the total population of nurses providing direct patient care in the hospital was manageable and accessible. The sample size was calculated based on the proportion from the previous study, using a 5% margin of error and a 95% confidence interval. A total of 400 nurses working across various clinical areas - inpatient wards, outpatient departments, critical care units, and operating theatres - were included in the study.

Inclusion and exclusion criteria

The present study included registered nurses employed full-time in providing direct care to their patients, who were able to read and write English or Tamil and were willing to participate in the study. The present study excluded staff nurses who worked at the managerial level and who were on an extended period of leave.

Tools and techniques

Data was collected using a self-report “BRIEF COPE inventory," which is a standardized instrument developed by Carver [11] to identify the coping approach adopted in stressful situations/events. It is an abbreviated version of the original COPE Inventory, consisting of 28 items organized into 14 subscales, each representing a distinct coping strategy. These subscales include active coping, planning, positive reframing, acceptance, humor, religion, use of emotional support, use of instrumental support, self-distraction, denial, venting, substance use, behavioral disengagement, and self-blame. Each item is rated on a 4-point Likert scale ranging from 1 (“I haven’t been doing this at all”) to 4 (“I’ve been doing this a lot”), indicating the frequency with which each coping behavior is used. The tool measures both adaptive (problem-focused and emotion-focused) coping strategies, such as active coping and positive reframing, as well as maladaptive coping mechanisms like denial and substance use. The Brief COPE has been shown to possess good internal consistency, test-retest reliability, and construct validity across diverse populations and cultural contexts, including healthcare professionals. It is particularly suited for studies exploring coping behaviors in stressful work environments, such as nursing, due to its brevity, clarity, and comprehensive coverage of coping dimensions. Researchers can analyze individual subscale scores or categorize coping into broader adaptive and maladaptive domains, depending on the study objectives.

Ethical consideration

The details of the study were explained using the subject information sheet, and informed consent was obtained before collecting the data. Prior to conducting the study, ethical clearance was obtained from the Institutional Ethics Committee. Nurses were not disturbed during their duty time. Each tool was explained clearly, including its purpose and direction in filling out the data.

Data collection and analysis

The data was collected from May 2019 to December 2019. Each tool was checked for completeness, and the data collection procedure terminated after thanking the participants. Collected data was coded and entered into an Excel spreadsheet (Microsoft Corp., Redmond, WA, USA), and the data was analyzed using R software (R Foundation for Statistical Computing, Vienna, Austria) (EZX-32 bit). Descriptive statistics were used to present the coping approaches, and inferential statistics were used to find the association between sociodemographic data and coping. The present study followed the STROBE checklist to report the study.

## Results

The mean age of the participants was 35±5 years, with a mean work experience of 10±5 years. Of them, 320 (80%) were working as regular nurses, and 320 (82%) worked on three shifts. Nurses reported an overall mean score of 61.6±12.6 on coping (Table 1).

**Table 1:** Coping strategies adopted by nurses.

Variables (Total scores)	Mean ± SD	Minimum score obtained	Maximum score obtained
Coping (28-112)	61.6±12.6	28	112

Nurses reported a maximum use of approach coping (27.8±4.4) than avoidance coping (22.6±4.1). Overall, out of 14 domains, religion (5.9±2.1) was the highest coping strategy reported by nurses, followed by behavioural disengagement (5.7±2), acceptance (5.3±2.2), and instrumental support (5±1.9) (Table 2).

**Table 2:** Mean, standard deviation of coping domain scores among nurses.

SL. No	Coping Domains score	Mean ± SD	Minimum score	Maximum score	Coping
1	Self-distraction (4)	4.3±1.8	2	8	Avoidance coping 22.6±4.1
2	Behaviour disengagement (4)	5.7±2	2	8	
3	Denial (4)	3.1±1.5	2	8	
4	Substance abuse (4)	2±0	2	4	
5	Venting (4)	3.2±1.7	2	8	
6	Self-blame (4)	4.3±1.8	2	8	
7	Active coping (4)	4.6±1.9	2	8	Approach coping 27.8 ± 4.4
8	Acceptance (4)	5.3±2.2	2	8	
9	Positive reframing (4)	3.7±1.8	2	8	
10	Emotional support (4)	4.8±1.8	2	8	
11	Instrumental support (4)	5±1.9	2	8	
12	Planning (4)	4.4±1.9	2	8	
13	Religion (4)	5.9±2.1	2	8	
14	Humour (4)	4.4±2	2	8	

Multiple linear regression analysis shows that marital status (β = 3.72, SE 3.05, p=<0.001) has 3.72 effect on coping while keeping other variables constant. (age β = - 0.04, R2 = 0.37, SE = 0.68, p<0.001; number of children β = -0.2, SE = 0.84, p<0.001; family income β = -0.19, SE = 0.86, p<0.001) (Table 3).

**Table 3:** Socio-demographic variables associated with coping among nurses.

Socio-demographic variables	N	Mean ± SD	Test Statistics	P-value	Regression coefficient / adjusted R^2^
	400				
Age (in years)					
a) <30	65	65±15			β = - 0.04
b) 31 to 35	97	61±11	F_(df,5)_ = 2.506	0.02*	R^2^ = 0.37
c) 36 to 40	76	61±10			P=<0.001
d) 41 to 45	72	62±7			SE = 0.68
e) 46 to 50	52	59±2			
f) >50	38	57±11			
Gender					
a) Male	12	62.1±13.8	t=0.143	0.886	-
b) Female	388	61.6±12.6	df=398		
Religion					
a) Christian	332	62±12			
b) Muslim	1	64±0	F_(df,2)_ = 0.479	0.62	-
c) Hindu	67	60±12			
Marital status					β = 3.72
a) Married	379	61.3±12.5	t=-2.086	0.037*	R^2^ = 0.37
b) Unmarried	21	67.2±13.3	df=398		P=<0.001
					SE= 3.05
Number of children					
a) Nil	49	67±15			β = -0.25
b) One	118	60±12	F_(df,3)_ = 3.17		R^2^ = 0.37
c) Two	198	61±11		0.02*	P=<0.001
d) Three and above	35	62±12			SE = 0.84
Residence					
a) Own house	315	61.7±12.2	t=0.356	0.721	-
b) Rented house	85	61.2±14.1	df=398		
Family type					
a) Joint family	108	61±12			
b) Nuclear family	290	62±12	F_(df,2)_ =0.57	0.5	-
c) Extended family	2	60±0			
Family dependent					
a) Nil	20	63±13			
b) One	4	54±8			
c) Two	97	60±11	F_(df,5)_ =0.75	0.585	
d) Three	40	63±16			-
e) Four	156	62±12			
Five and above	83	62±12			
Mode of transport					
a) By walk	29	62±18			
b) Public Vehicle	281	61.6±12	F_(df,2)_ =0.071	0.9	-
c) Own Vehicle	90	61.3±12			
Distance from house to hospital					
a) <5 km	105	63±13			
b) 5-10 km	93	60±12			
c) 11-15 km	60	63±12	F_(df,4)_ =1.24	0.29	-
d) 15-20 km	45	64±11			
e) >20 km	97	59±12			
Monthly income (In Rupee)					
a) <5000	13	78±18			β = -1.5
b) 5001-10000	99	64±12			R^2^ = 0.37
c) 10001 to 15000	74	61±12	F_(df,5)_ =6.32		P= <0.001
d) 15001 to 20000	30	65±14		<0.001***	SE =0.88
e) 20001 to 25000	18	58±8			
f) >25000	166	59±11			
Family income (In Rupee)					
a) <5000	1	40±0			
b) 5001-10000	3	67±6			β = -0.19
c) 10001 to 15000	15	70±21	F_(df,5)_ =3.25	0.006**	R^2^ = 0.37
d) 15001 to 20000	13	60±13			P= <0.001
e) 20001 to 25000	60	65±12			SE= 0.86
f) >25000	308	61±11			

Multiple linear regression analysis shows that job type has 2.2 effect (β = 2.20, SE = 0.9, p <0.001) on coping while keeping other variables constant, years of experience (β = -0.96, SE = -1.87, p <0.001) (Table 4).

**Table 4:** Professional variables associated with coping among nurses.

Professional variables	N	Mean ± SD	Test Statistics	P-value	Regression coefficient / adjusted R^2^
	400				
Educational qualification					
a) GNM	368	62±12			
b) BSc Nursing	28	63±11	F_(df,2)_ =1.91	0.14	-
c) MSc Nursing	4	50±16			
Area of work					
a) ICU	54	66.2±12			
b) Medical ward	59	61.01±14			
c) OPD	59	63.2±12			
d) Surgical ward	42	62±11.6	F_(df,7)_ = 1.81	0.08	-
e) OT	38	60.2±12.5			
f) Labour/Gynaec	48	58.8±14.6			
g) Emergency	63	60.7±11.4			
h) Others	37	59.4±10.6			
Years of experience					
a) <2 yrs	17	65±14			β = -0.96
b) 2-5 yrs	28	69±16			R^2^ = 0.36
c) 6-10 yrs	101	62±11	F_(df,5)_=2.704	0.02*	P= <0.001
d) 11-15 yrs	123	60±12			SE = -1.87
e) 16-20 yrs	54	61±10			
f) >20 yrs	77	61±12			
Shift of duty					
a) 2 shifts	17	58±12	F_(df,2)_ =0.736	0.4	
b) 3 shifts	370	62±12	Df=2		-
Single/general shift	13	60±6			
Work hours per week (average including night duty)			t=-0.644	0.52	
a) 36 - 48 hours	343	61.4±13.1	df=398		
b) 49 - 72 hours	57	62.6±9.6			-

## Discussion

This moderate coping score in the study suggests that while nurses demonstrate an ability to cope with workplace stress, there remains room for improvement. Coping mechanisms are crucial in mitigating the negative effects of occupational stress, particularly in high-stress environments such as healthcare [12]. Variability in coping scores, ranging from 28 to 112, highlights differences in individual resilience and access to coping resources. Similar findings were reported by Sharma et al., who found that nurses with higher coping scores utilized proactive strategies such as seeking support and problem-solving, while lower scores were associated with avoidance or emotional disengagement [13].

The moderate coping scores align with existing literature suggesting that nurses often rely on emotion-focused strategies, such as maintaining optimism and self-regulation, to manage stressors [14]. However, the data also underscore the need for structured interventions to enhance coping abilities. Training programs focused on stress management and resilience-building have been shown to improve coping skills and reduce stress levels among nurses [15]. Additionally, organizational support, such as counselling services and supportive leadership, plays a pivotal role in enhancing coping mechanisms [16]. Addressing barriers to effective coping, such as workload and resource limitations, could further empower nurses to handle occupational stress more effectively. As nurses demonstrate moderate coping abilities, targeted interventions and supportive organizational policies are crucial to enhance their resilience and ensure better mental well-being.

The findings demonstrate that nurses reported greater use of approach coping strategies (27.8 ± 4.4) compared to avoidance coping strategies (22.6 ± 4.1). This indicates that nurses are more likely to engage in proactive strategies, such as problem-solving and seeking social support, to manage workplace stress. Approach coping, which involves actively addressing stressors, has been associated with better psychological outcomes and resilience among healthcare professionals [15]. In contrast, avoidance coping, which includes behaviours such as denial or disengagement, may offer temporary relief but is generally linked to poorer mental health outcomes [12]. Among the 14 domains of coping assessed, religion emerged as the most frequently used strategy (5.9 ± 2.1). The reliance on religious coping is consistent with previous studies that highlight the importance of spirituality and faith in reducing stress among nurses, particularly in high-stress environments [17]. Religious practices, such as prayer or meditation, provide emotional support, instill hope, and offer a sense of control in situations perceived as overwhelming [13]. However, while religious coping can be beneficial, it may not address the root causes of stress, emphasizing the need for complementary strategies.

Behavioral disengagement (5.7 ± 2.0) was the second most commonly reported coping strategy. This finding aligns with research suggesting that disengagement is often employed as a response to persistent stressors that seem unmanageable [14]. While it may offer short-term relief, excessive reliance on disengagement can lead to burnout and decreased job performance, particularly in high-stakes professions like nursing. Acceptance (5.3 ± 2.2) was also frequently reported as a coping mechanism, reflecting nurses’ acknowledgment of stressful situations and their ability to adapt to them. Acceptance is considered an adaptive strategy that helps individuals maintain emotional stability by focusing on aspects within their control [18]. This strategy is particularly relevant in nursing, where unpredictable and emotionally taxing situations are common. Instrumental support (5.0 ± 1.9) was another significant coping domain identified. Nurses often rely on colleagues, supervisors, and institutional support systems to manage work-related stress. Social support has been consistently linked to lower stress levels and improved job satisfaction in nursing populations [16]. Peer support, in particular, fosters a sense of camaraderie and reduces feelings of isolation, contributing to better overall coping. The preference for approach coping over avoidance coping and the frequent use of adaptive strategies like acceptance and instrumental support highlight the resilience of nurses in managing workplace stress. However, the prominence of behavioural disengagement suggests that some nurses may struggle to effectively address chronic stressors, underscoring the need for targeted interventions.

Organizations can play a critical role in promoting adaptive coping strategies by providing stress management training, fostering supportive work environments, and ensuring access to mental health resources [19]. Additionally, spiritual care programs that align with the religious practices of nurses may further enhance their coping capabilities [17]. Nurses demonstrate a commendable ability to cope with stress through approach strategies and adaptive mechanisms. Addressing the underlying causes of stress and providing comprehensive support systems are essential to sustain their well-being and performance in the long term. The study's findings indicate a significant association between socio-demographic variables and coping strategies among nurses. Specifically, age, number of children, monthly income, total family income, and marital status were found to influence coping behaviors significantly. These findings align with existing research suggesting that socio-demographic factors play a crucial role in shaping coping mechanisms among healthcare professionals [20]. Age was found to significantly influence coping strategies (F = 2.50, p = 0.02). Older nurses may develop better coping skills through accumulated experience and exposure to stress, as suggested by Lim et al. [8]. Conversely, younger nurses may face challenges in managing stress due to limited professional experience, necessitating targeted interventions to enhance coping skills in early career stages. The number of children also emerged as a significant factor (F = 3.17, p = 0.02), which is consistent with studies highlighting the dual burden of work and family responsibilities for nurses [21]. Nurses with more children may experience greater demands, leading to the use of adaptive coping strategies to balance work and personal life.

Income-related variables, including monthly income (F = 6.32, p < 0.001) and total family income (F = 3.25, p = 0.006), showed significant associations with coping. Higher income levels may provide better access to resources that reduce stress, such as professional support or recreational activities [4]. Marital status was another significant determinant (t = -2.08, p = 0.037). Married nurses were found to adopt more effective coping strategies, potentially due to spousal support and shared responsibilities, which mitigate stress [22]. This was further supported by the multiple linear regression analysis, where marital status had the highest effect on coping (β = 3.72, SE = 3.05, p < 0.001), suggesting its critical role in shaping coping behaviours. Overall, these findings highlight the importance of considering socio-demographic factors in designing interventions to improve coping strategies among nurses. Targeted support, such as flexible working hours for nurses with children or financial counselling for those with low income, may enhance their resilience and well-being. This study highlights the significant associations between professional variables, such as job pattern and years of experience, and coping strategies adopted by nurses. The findings are consistent with existing literature emphasizing the impact of professional roles and tenure on stress management and coping mechanisms in healthcare settings [23]. Job pattern was significantly associated with coping (F = 6.2, p = 0.002). Nurses with more stable or predictable job patterns may employ adaptive coping strategies more effectively than those in rotating or irregular shifts, which are linked to higher stress levels and reduced coping capacities. Evidence from Ayala and Carnero suggests that shift work disrupts circadian rhythms, contributing to job stress and less effective coping strategies [24]. Years of experience also showed a significant association with coping (F = 2.70, p = 0.02). Nurses with more experience tend to develop refined coping mechanisms due to familiarity with job demands and challenges. This finding is supported by Ofei et al., who observed that experienced nurses were more likely to employ problem-focused coping strategies compared to their less experienced counterparts [25]. However, the regression analysis revealed a negative association (β = -0.96, SE = -1.87, p < 0.001), indicating that while experience fosters coping, it might also lead to emotional exhaustion over time if workplace stressors are not mitigated. The regression analysis also demonstrated that job type significantly influenced coping (β = 2.20, SE = 0.9, p < 0.001). Nurses in specialized or administrative roles may adopt more effective coping strategies due to structured workflows or reduced patient load compared to frontline nurses in high-intensity environments [26]. These findings emphasize the need for targeted workplace interventions, such as workload adjustments, mentorship programs for less experienced nurses, and organizational support for nurses in high-stress job patterns, to promote effective coping mechanisms.

Strengths & limitations

The study offers insights into coping strategies employed by nurses in diverse workplace settings (war/ICU/OPD/OT). A sample size of 400 nurses ensures robust data and improves generalizability. Total enumerating sampling reduces selection bias, and inclusion criteria focused on direct patient care enhance relevance to frontline nursing challenges. Self-reported data may be prone to social desirability bias.

## Conclusions

Strengthening coping capacities for nurses requires a multidimensional approach that combines individual resilience-building with systemic organizational reforms. Institutions should implement structured stress management programs, including mindfulness-based stress reduction, relaxation training, and peer-support groups, to promote psychological well-being. Establishing counseling and employee assistance services can provide confidential support and guidance for stress management. From an educational perspective, integrating coping and resilience modules into nursing curricula can empower future nurses with adaptive strategies before entering the workforce. Periodic workshops, simulation-based sessions, and reflective practices can enhance emotional intelligence and self-efficacy in coping. Organizational strategies - such as adequate nurse-patient ratios, clear job descriptions, participatory decision-making, and recognition of nurses’ contributions - are also essential to mitigate occupational stress. Culturally, incorporating holistic approaches such as yoga, meditation, and spirituality-based coping, which resonate with Indian values, may further enhance resilience. Collaboration between administrators, educators, and policy-makers is vital to developing sustainable mental health and wellness programs for nurses. By adopting these evidence-based and context-sensitive interventions, healthcare institutions can foster a supportive work environment, improve nurses’ well-being, and ensure higher quality of patient care.
